# Runx1 regulates zebrafish neutrophil maturation *via* synergistic interaction with c-Myb

**DOI:** 10.1016/j.jbc.2021.100272

**Published:** 2021-01-09

**Authors:** Zhibin Huang, Kemin Chen, Yali Chi, Hao Jin, Li Li, Wenqing Zhang, Jin Xu, Yiyue Zhang

**Affiliations:** 1 Division of Cell, Developmental and Integrative Biology, School of Medicine, South China University of Technology, Guangzhou, P.R. China; 2 Department of Developmental Biology, School of Basic Medical Sciences, Southern Medical University, Guangzhou, P.R. China; 3 State Key Laboratory of Molecular Neuroscience, Division of Life Science, The Hong Kong University of Science and Technology, Hong Kong, P.R. China; 4 The Key Laboratory of Freshwater Fish Reproduction and Development, Ministry of Education, State Key Laboratory Breeding Base of Eco-Environments and Bio-Resources of the Three Gorges Area, School of Life Science, Southwest University, Chongqing, P.R. China

**Keywords:** *runx1*, *c-myb*, neutrophil, zebrafish, AD, activation domain, ChIP, chromatin immunoprecipitation, Co-IP, coimmunoprecipitation, DBD, DNA-binding domain, dpf, days postfertilization, hpf, hours postfertilization, ID, inhibitory domain, Lyz, lysozyme C, Mpx, myeloperoxidase, Npsn, nephrosin, NRD, negative-regulatory domain, RHD, RUNT homology domain, SB, Sudan black B, SEM, standard error of the mean, Srgn, serglycin, TAD, transactivation domain, VE DIC, video-enhanced differential interference contrast, WISH, whole mount *in situ* hybridization

## Abstract

Neutrophils play an essential role in the innate immune defense system in vertebrates. During hematopoiesis, the full function of neutrophils involves maturation of granules and related enzymes. Yet, transcription regulators that promote neutrophil maturation remain largely undefined. Here, two hematopoiesis-defective zebrafish mutants, *runx1*^*w84x*^ and *c-myb*^*hkz3*^, were used to investigate the *in vivo* roles of Runx1 in cooperation with c-Myb in regulating neutrophil maturation. Loss of *runx1* impairs primitive neutrophil development. Additional regulation of *c-myb*^*+/−*^ and *c-myb*^*−/−*^ induces a more severe phenotypes suggesting a synergistic genetic interaction between *c-myb* and *runx1* in neutrophil maturation. Further studies revealed that the two transcription factors act cooperatively to control neutrophil maturation processes *via* transactivating a series of neutrophil maturation-related genes. These data reveal the *in vivo* roles of Runx1 in regulating primitive neutrophil maturation while also indicating a novel genetic and molecular orchestration of Runx1 and c-Myb in myeloid cell development. The study will provide new evidence on the regulation of neutrophil maturation during hematopoiesis.

Neutrophils are the most abundant phagocytes essential for the first line of defense in the innate immune system. Mature neutrophils play an important role in pathogen clearance, response to tissue injury, and in mediating the inflammatory response (1). Dysregulation of granulopoiesis can cause neutropenia, myeloid leukemia, or neutrophil function disorders (2, 3, 4, 5, 6, 7). Thus, understanding how neutrophils differentiate into functional mature cells might facilitate the development of new therapeutic strategies for the treatment of neutrophil-related disorders.

The function of mature neutrophils requires the development of characteristic neutrophil granules along with stored enzymes. These neutrophil-specific elements are formed at different stages during neutrophil maturation. Mammalian neutrophils contain four types of granules: azurophil granules, specific granules, gelatinase granules, and secretory granules. Each subtype of granule contains highly specific storage proteins that carry out different immune functions (8). Neutrophil granule subtypes are released in order to lyse and eradicate microbes when neutrophils are activated during infections (2). Digestive enzymes are key components in neutrophil granules. For example, lysozyme C (Lyz), is a key bactericidal enzyme found in all types of neutrophil granules, and myeloperoxidase (Mpx) is an abundant peroxidase stored in neutrophil azurophilic granules (2). Sorting and packing neutrophil granule proteins by proteoglycans, such as serglycin (Srgn), are essential for neutrophil differentiation (9, 10). Neutrophil maturation requires that these granule-related proteins are properly produced, yet the molecular basis controlling the process remains largely unknown.

Several hematopoietic-specific transcription factors are reported to control neutrophil granule-related protein expression. RUNX1 has been described as a pivotal transcription factor during definitive hematopoiesis (11, 12, 13). In neutrophil development, Runx1 is reported to promote granulocytic over monocytic lineage fate choice in zebrafish (14). Yet, the function of RUNX1 in neutrophil differentiation and maturation is still debatable. It has been reported that RUNX1 could regulate *Mpx* and *Elane* transcription in myeloid cell lines (15, 16, 17). Similarly, recent mouse data demonstrated that RUNX1-haploinsufficient hematopoietic progenitors impaired *in vitro* differentiation in neutrophils by repressing *Cebpe* expression (18). However, another study found that conditional ablation of the *Runx1* gene in adult mice paradoxically expands myeloid pools to an extent without incurring any discernible differentiation blockage (19). Therefore, whether RUNX1 plays roles in granulocyte differentiation and maturation *in vivo* is still unclear, especially in early developmental stages. We previously showed that *lyz* is a direct target of c-Myb in regulating neutrophil maturation (20). Interestingly, *lyz* is also a transcriptional target of Runx1 (21); however, whether RUNX1 participates in neutrophil differentiation and maturation lacks sufficient *in vivo* evidence. Furthermore, whether the neutrophil maturation process is achieved by the orchestration of these two transcription factors requires genetic verification.

Here, we used two hematopoietic-defective zebrafish mutants, *runx1*^*w84x*^ (22) and *c-myb*^*hkz3*^ (23) to determine the role of Runx1 during neutrophil maturation. These mutants were used to elucidate the genetic interaction of the two transcription factors through genetic epistasis and biochemical analysis. It was found that Runx1 cooperates with c-Myb to control neutrophil maturation in zebrafish embryonic myelopoiesis. This study elucidates the genetic networks that orchestrate primitive myeloid cell development, improving our understanding of the pathogenesis of neutrophil-related diseases.

## Results

### Runx1 regulates primitive neutrophil maturation

**Figure 1:**
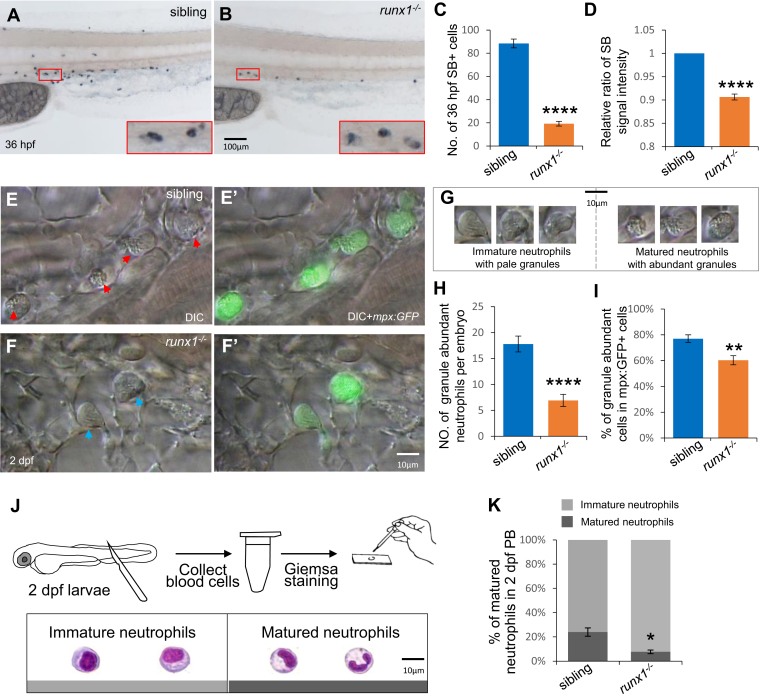
**Neutrophil maturation was affected by *runx1***^***w84x***^**mutation.***A*–*D*, SB staining showed decrease number and intensity of SB^+^ cells in 36-hpf *runx1*^*−/−*^ mutants (*B*) compared with siblings. *Red boxes* show enlarged detail of SB^+^ cells in each group (×4). *C*, quantification of numbers of 36-hpf SB^+^ cells (each n ≥ 22). Neutrophils were counted based on the SB signals of the whole embryos. *D*, relative ratio of SB signal intensity was calculated in *runx1* mutants and siblings. *E*–*H*, granule status in mpx:GFP^+^ neutrophils. (*E*, *F*, *E’* and *F’*) *In vivo* VE DIC microscopy revealed reduction of granules in neutrophils in 2-dpf *runx1* mutants (*F* and *F’*) compared with siblings (*E* and *E’*) in Tg(*mpx*:*GFP*) background (each n ≥ 22). Left panels are bright field DIC images. *Right panels* are overlays of bright field DIC images with corresponding GFP fluorescent images. *Red arrowheads* indicate matured neutrophils. *Blue arrowheads* indicate immature neutrophils. *G*, representative images of matured neutrophils with abundant granules and immature neutrophils with pale granules. *H* and *I*, quantification of absolute numbers of granule-abundant neutrophils per embryo (*H*) and relative percentage of granule-abundant cells in total mpx:GFP^+^ neutrophils (*I*). *J* and *K*, May–Grünwald–Giemsa staining of neutrophils in 2-dpf embryos (*J*) and neutrophils were quantitated by morphology (*K*). *Gray* and *dark gray* represent immature and mature neutrophils by their nucleic morphology. Scale bars, 100 μm (*A* and *B*), and 10 μm (*E*, *F*, *E’*, *F’*, *G*, and *J*).

Mature neutrophils are characterized by abundant granules in the cytoplasm, which can be specifically stained by Sudan Black B (SB) (14, 24). Similar to the phenotype of *c-myb*^*−/−*^ mutants in primitive myelopoiesis (20), *runx1*^*−/−*^ mutants had reduced the number of SB^+^ neutrophils at 36 h postfertilization (hpf) (14) (Fig. 1, *A*–*C*). More importantly, the signal intensity for SB staining of the neutrophils was lower in mutants compared with that in siblings (Fig. 1, *A*, *B* and *D*), suggesting that Runx1 is involved in neutrophil maturation. Since mature neutrophils have abundant granules in their cytosol, video-enhanced differential interference contrast (VE DIC) (25) microscopy was used to observe neutrophil granule status and abundance in live embryos at 2 days postfertilization (dpf) in Tg(mpx:GFP) background. GFP was expressed mainly in neutrophils under the control of the neutrophil-specific promoter (26). Although mpx:GFP^+^ cell numbers were decreased and the expression was weakened in the mutants, we could still catch the remaining mpx:GFP^+^ neutrophils under the microscopy. Indeed, *runx1*^*−/−*^ mutants contained fewer mature neutrophils than observed in siblings (Fig. 1, *E*, *F*, *E’*, *F’* and *G*–*I*). Mature neutrophils were also characterized by banded and segmented nuclei (20, 27). Therefore, mature and immature neutrophils were quantified by staining nuclei with May–Grünwald–Giemsa stain. These studies showed a significant decrease in mature neutrophils in 2-dpf *runx1*^*−/−*^ mutants compared with siblings (Fig. 1, *J* and *K*). The data demonstrate that primitive neutrophils were affected by the *runx1* mutation in zebrafish.

### Runx1 regulates neutrophil maturation accompanying c-Myb

**Figure 2:**
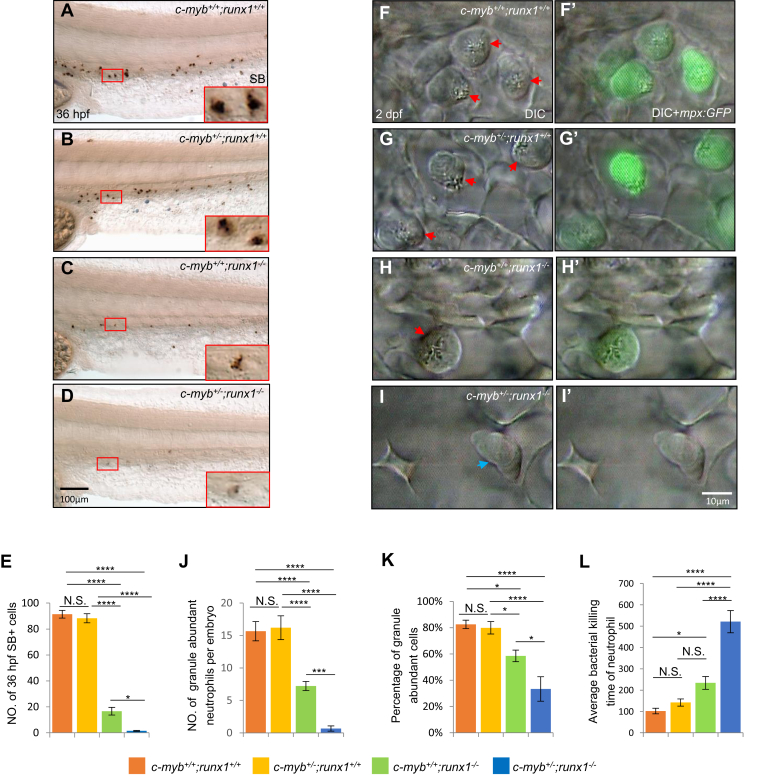
**c-Myb and Runx1 synergistically regulate neutrophil maturation.***A*–*E*, SB staining showed further decrease in intensity and number of SB^+^ cells in 36-hpf *c-myb*^*+/−*^;*runx1*^*−/−*^ double mutants (*D*) compared with single mutants. *Red boxes* show enlarged detail of SB^+^ cells in each group (×4). *E*, quantification of numbers of 36-hpf SB^+^ cells (each n ≥ 18). (*F*–*I* and *F’*–*I’*) *In vivo* VE DIC microscopy revealed further reduction of granules in neutrophils in 2-dpf double mutants (*I* and *I’*) compared with single mutants (*G*, *H*, *G’* and *H’*) in Tg(*mpx*:*GFP*) background (each n ≥ 15). *Red arrowheads* indicate matured neutrophils with abundant granules. *Blue arrowheads* indicate immature neutrophils with pale granules. *J*–*K*, quantification of absolute numbers of granule abundant-neutrophils per embryo (*J*) and relative percentage of granule-abundant cells in total mpx:GFP^+^ neutrophils (*K*), each n ≥ 15. Scale bars equal 10 μm. *L*, neutrophil bacterial clearance time in each group. Scale bars, 100 μm (*A*–*D*), and 10 μm (*F*–*I* and *F’*–*I’*).

To determine the role of *runx1* in relation with *c-myb* in neutrophil maturation, a genetic approach was used to compare the neutrophil phenotypes of single *runx1* or *c-myb* mutants with composite mutants derived from crossing *c-myb*^*+/−*^;*runx1*^*+/−*^ double heterozygotes. Since *c-myb*^*+/−*^ heterozygous embryos were indistinguishable from wild-type embryos in the number of SB^+^ cells at 36 hpf (20), we focused on the phenotype comparisons of *c-myb*^*+/−*^;*runx1*^*−/−*^ double mutants and *c-myb*^*+/−*^ and *runx1*^*−/−*^ single mutants to determine whether additional *c-myb*^*+/−*^ heterozygotes would yield more severe phenotypes. When one allele of *c-myb*^*+/−*^ was introduced into *runx1*^*−/−*^, the number of SB^+^ cells was further reduced (Fig. 2, *A*–*E*). Consistently, the *c-myb*^*−/−*^;*runx1*^*−/−*^ double homozygotes had nearly no SB^+^ cells left (data not shown). Furthermore, the DIC microscopy of live embryos (Fig. 2, *F*–*I*, *F’*–*I’* and *J*–*K*) showed that the residual neutrophils in *c-myb*^*+/−*^;*runx1*^*−/−*^ (Fig. 2, *I* and *I’*) elicited more immature characteristics, with scarce granules almost lacking mpx:GFP expression when compared with *c-myb*^*+/−*^;*runx1*^*+/+*^ (Fig. 2, *G* and *G’*) or *c-myb*^*+/+*^;*runx1*^*−/−*^ mutants (Fig. 2, *H* and *H*’). To determine whether the synergistic regulation by c-Myb and Runx1 has biological consequences on neutrophil function, we examined the rate of bacterial killing by neutrophils in each group. As expected, additive *c-myb*^+/−^ further attenuated the *runx1*^*−/−*^ bacterial killing activity, as *c-myb*^+/−^;*runx1*^*−/−*^ mutants prolonged the bacterial clearance time compared with *runx1*^*−/−*^ single mutants (Fig. 2*L*). The above data suggest that *runx1* could genetically cooperate with *c-myb* to potentiate neutrophil maturation.

### Neutrophil maturation-related genes were coregulated by Runx1 and c-Myb

**Figure 3:**
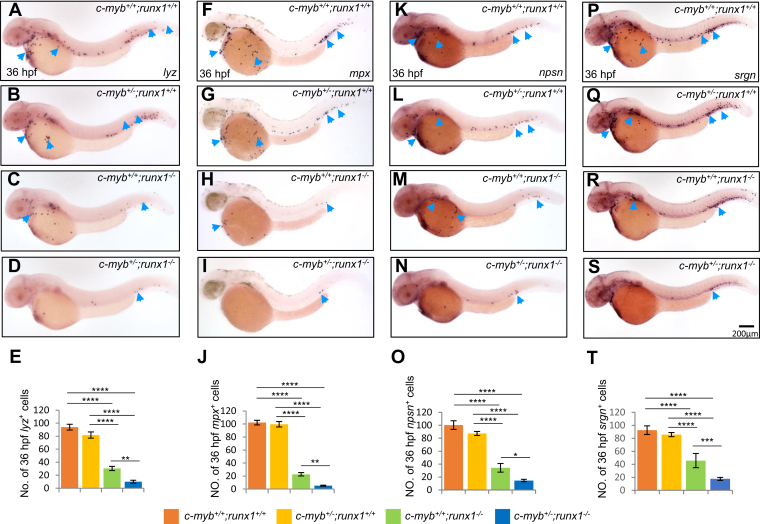
**Genetic interaction between *c-myb* and *runx1* on neutrophil-specific genes.***A*–*D*, WISH showed further decrease of *lyz* expression in 36-hpf double mutants (*D*) compared with single mutants (*B* and *C*). *E*, quantification of numbers of 36-hpf *lyz*^+^ cells (each n ≥ 16). *F*–*J*, WISH showed further decrease of *mpx*^+^ cells in 36-hpf double mutants (*I*) compared with single mutants. *J*, quantification of numbers of 36-hpf *mpx*^+^ cells (each n ≥ 20). *K*–*N*, WISH showed further decrease of *npsn*^+^ cells in 36-hpf double mutants (*N*) compared with single mutants. *O*, quantification of numbers of 36-hpf *npsn*^+^ cells (each n ≥ 8). *P*–*S*, WISH showed further decrease of *srgn*^+^ cells in 36-hpf double mutants (*S*) compared with single mutants. *T*, quantification of numbers of 36-hpf *srgn*^+^ cells (each n ≥ 8). *Blue arrowheads* indicate *lyz*^+^, *mpx*^+^, *srgn*^+^, and *npsn*^+^ neutrophils in each row. Scale bars, 200 μm.

Similar to mammals, zebrafish neutrophil maturation also required typical neutrophil granule-related components and digestive enzymes, such as Lyz, Mpx, and nephrosin (Npsn), to be properly produced (2, 28). It has been reported that zebrafish *lyz* is directly targeted by c-Myb (20) and transcriptionally regulated by Runx1 (21), but whether *lyz* could be coactivated by c-Myb and Runx1 *in vivo* is unclear. To test the combined effects, whole mount *in situ* hybridization (WISH) was performed to compare *lyz* expressions between *c-myb*^*+/−*^;*runx1*^*+/−*^ crossed embryos. Our laboratory previously showed that *c-myb*^*−/−*^ homozygotes had almost no *lyz*^*+*^ cells (20) (Fig. S1, *A* and *A’*), that the signal numbers between *c-myb*^*+/−*^;*runx1*^*+/+*^ and *c-myb*^*+/+*^;*runx1*^*+/+*^ were comparable (Fig. 3, *A* and *B*). These results indicate that one *c-myb* allele deficiency (*c-myb*^*+/−*^ heterozygous) does not affect *lyz*^*+*^ signals when the *runx1* gene is not mutated. In *c-myb*^*+/+*^;*runx1*^*−/−*^ embryos (Fig. 3*C*), the *runx1*^*−/−*^ homozygous mutation decreased *lyz*^*+*^ cells to one-third of those in *c-myb*^*+/+*^;*runx1*^*+/+*^ embryos (Fig. 3*A*). Importantly, the inadequate *lyz*^*+*^ cells in *runx1*^*−/−*^ background mutants were further reduced when one allele of *c-myb*^*+/−*^ was introduced, as the average *lyz*^*+*^ cell number per embryo was decreased from 30 in *c-myb*^*+/+*^;*runx1*^*−/−*^ (Fig. 3*C*) to 10 in *c-myb*^*+/−*^;*runx1*^*−/−*^ (Fig. 3, *D* and *E*). The above data suggest a synergistic regulation of the two transcription factors on the expression of *lyz*. Since neutrophil maturation requires proper sequential expression of digestive enzymes, it was hypothesized that the synergistic effects of *c-myb* and *runx1* are likely for *lyz*, as well as for other related genes. Zebrafish Mpx and Npsn are both neutrophil-specific granzymes used for host clearance of microbe infections (28, 29). Similarly, both *mpx*^*+*^ and *npsn*^*+*^ cells were further reduced when one or two alleles of the *c-myb* mutation were introduced in *runx1*^*−/−*^ mutants (Fig. 3, *F*–*O*, and Fig. S1, *B*, *C*, *B’* and *C’*). Moreover, the granule-formation-related glycoprotein-*srgn* was greatly downregulated in *c-myb* zebrafish mutants (20), suggesting *srgn* is also regulated by c-Myb. Expectedly, in *c-myb*^*+/−*^;*runx1*^*−/−*^ double mutants, *srgn*^*+*^ cells showed a greater inhibition compared with *c-myb*^*+/−*^ or *runx1*^*−/−*^ single mutants (Fig. 3, *P*–*T* and Fig. S1, *D* and *D’*). Consistently, the *c-myb*^*−/−*^;*runx1*^*−/−*^ double mutants showed the least amount of expression of those genes (Fig. S1, *D* and *D’*). The above data suggest that there is a synergistic regulation of *c-myb* and *runx1* on downstream neutrophil maturation-related genes. Taken together, these results indicate that Runx1, cooperatively with c-Myb, is essential for neutrophil maturation by positively regulating neutrophil maturation-related genes.

### c-Myb and Runx1 physically interact to directly promote neutrophil-specific genes transcription

**Figure 4:**
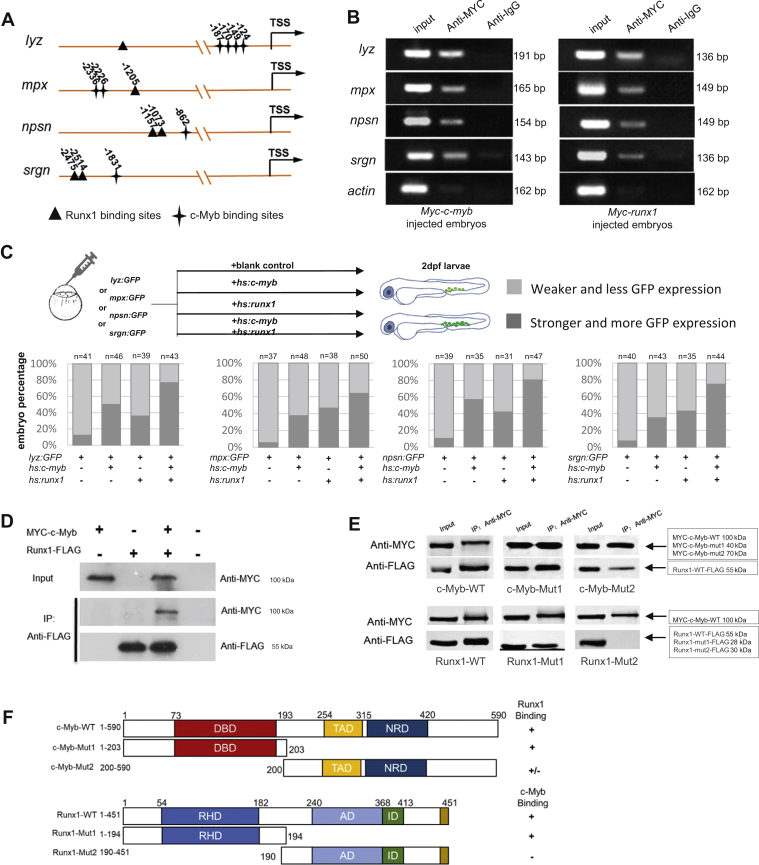
**c-Myb and Runx1 synergistically regulate neutrophil-specific genes transcription.***A*, schematic diagram of the *lyz*, *mpx*, *srgn*, and *npsn* promoter region. The transcription starting site is designated as TSS. Putative c-Myb and Runx1 binding sites are marked by *stars* and *triangles* respectively using JASPAR online software. *B*, ChIP shows that MYC-tagged c-Myb and MYC-tagged Runx1 bind to the promoter region of the *lyz*, *mpx*, *npsn*, and *srgn* promoters. Lysates from the embryos injected with the *Myc-c-myb* (*left panel*) and *Myc-runx1* plasmids (*right panel*) were precipitated with anti-MYC antibodies. The precipitates were then subjected to semiquantitative PCR analysis compared with anti-IgG control. Input DNA control was on the left of each panel. *C*, *Upper panel* shows representative procedures of GFP reporter assay. *lyz*(-2.4k):*GFP*, *mpx*(-8k):*GFP*, *npsn*(-2k):*GFP and srgn*(-5k)*:GFP* coinjected with or without *hs:c-myb*, *hs:runx1* plasmid. Two groups were classified by GFP fluorescence intensity and GFP^+^ cells number. *Lower panel* shows embryo percentage of each group (each n ≥ 20). *Gray* represents weaker GFP expression and less GFP+ cells. *Dark gray* represents stronger GFP expression and more GFP+ cells. *D*, *in vitro* coimmunoprecipitation experiment detected the interaction between c-Myb and Runx1. *Myc*-tagged *c-myb* and *Flag*-tagged *runx1* were transfected into 293T cells as indicated and cell lysates were immunoprecipitated with anti-FLAG antibody. The immunoprecipitants were examined by western blot using anti-MYC and anti-FLAG antibodies. Input represents 10% of total cell lysates used for immunoprecipitation. *E*, deletions impinging on the c-Myb DBD (*top panel*) and Runx1 RHD (*low panel*) decrease the interaction between c-Myb and Runx1. MYC-c-Myb was immunoprecipitated, and western blots were probed with antibodies to MYC or FLAG. *F*, summary of c-Myb and Runx1 mapping experiments. AD, activation domain; DBD, DNA-binding domain; ID, inhibitory domain; NRD, negative-regulatory domain; RHD, RUNT-homology domain; TAD, transactivation domain.

To determine whether the four genes (*lyz*, *mpx*, *npsn*, and *srgn*) are directly targeted by c-Myb and Runx1, the regulatory regions of these genes were analyzed. It was found that these genes have putative c-Myb and Runx1 binding sites (Fig. 4*A*). Chromatin immunoprecipitation polymerase chain reaction (ChIP-PCR) assays were performed to ascertain whether the two transcription factors can directly bind to the putative sites *in vivo*. To address this question, embryos were injected with *Myc*-tagged *c-myb* or *runx1* plasmids to overexpress c-Myb and Runx1 for ChIP-PCR analysis in zebrafish. The results showed that all putative binding sites of the target genes were coprecipitated using an anti-MYC antibody (Fig. 4*B*). This result demonstrates that these neutrophil-specific genes are all directly regulated by c-Myb and Runx1 respectively.

To further support the hypothesis, the *in vivo* GFP reporter assay was performed by expressing neutrophil-specific reporter constructs together with *c-myb* or *runx1* overexpression plasmids in zebrafish embryos. As reported, by using the zebrafish reporter assay c-Myb overexpression increased *lyz* promoter transcription activity (20). In the present report, it was also found that c-Myb overexpression increased *mpx*, *npsn*, and *srgn* promoter transcription activity (Fig. 4*C*). Compared with overexpressing c-Myb only, transcription activation of the four target genes were all enhanced by Runx1 coexpression (Fig. 4*C*). This suggests a cooperation of c-Myb and Runx1 on targeting downstream genes.

Next, it was asked whether c-Myb and Runx1 physically interact in these molecular functions. Coimmunoprecipitation (Co-IP) experiments were performed in 293T cells cotransfected with *runx1-Flag* and *Myc*-*c-myb* constructs. Cell lysates were immunoprecipitated using an anti-FLAG antibody, followed by western blot analysis using anti-MYC and anti-FLAG antibodies. Immunoprecipitating Runx1-FLAG resulted in coprecipitation of MYC-c-Myb (Fig. 4*D*). This suggests protein-level cooperation between zebrafish Runx1 and c-Myb. In addition, the co-IP assay was performed in zebrafish embryos. The result was consistent with that observed in the 293T cells (Fig. S2). To identify the regions of c-Myb and Runx1 that are necessary for their interaction, zebrafish c-Myb and Runx1 proteins were further truncated for the co-IP assay. The results showed that the DNA binding domain (DBD) of c-Myb and Runt homologs domain (RHD) of Runx1 are predominantly responsible for the observed interaction (Fig. 4, *E* and *F*). However, the constructs for overexpressing truncated c-Myb or Runx1 proteins were injected into *c-myb*^*hkz3*^ and *runx1*^*w84x*^ mutants respectively, but the overexpression could not rescue the neutrophil defects in these two mutants (data not shown). These data suggest that the truncated proteins may also affect the essential functions of these proteins, such as DNA binding activity.

The final experiment was designed to explore whether there exists a mutual dependency on *c-myb* and *runx1* expression in early neutrophil progenitors. Expression levels were checked at 18 hpf, a crucial stage in embryogenesis when zebrafish myelopoiesis arises directly from the rostral blood island (RBI) (24, 30). In the RBI region, *c-myb* expression was found to be unchanged in *runx1*^*−/−*^ embryos (Fig. S3, *A* and *B*), and *runx1* expression was also unaltered in *c-myb*^*−/−*^ embryos (Fig. S3, *C* and *D*). These expression data suggest that *c-myb* and *runx1* are transcriptionally independent of each other during early neutrophil development.

**Figure 5:**
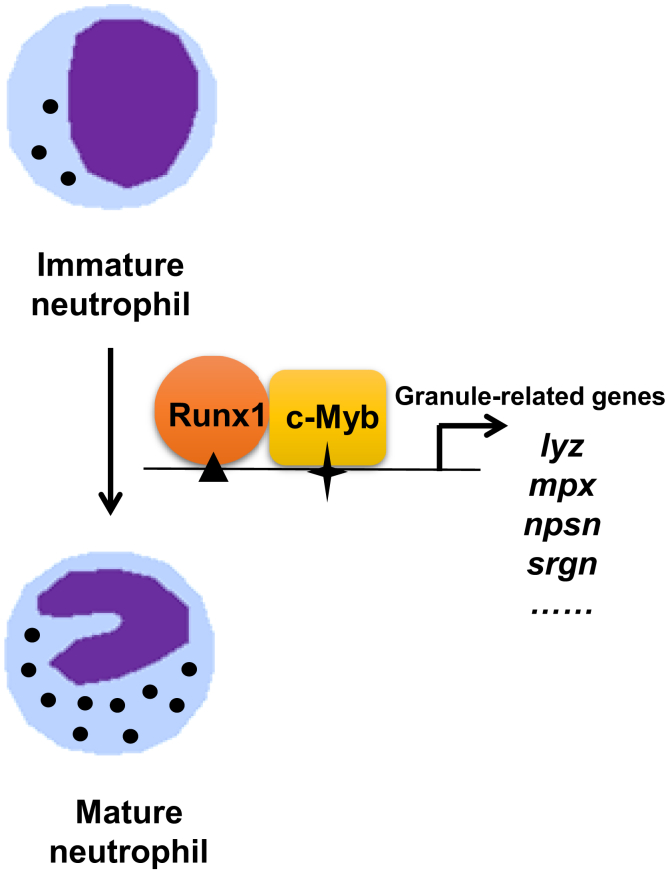
**Models of neutrophil regulation by c-Myb and Runx1 of in zebrafish.** Synergistic interaction of c-Myb and Runx1 in neutrophil maturation. c-Myb and Runx1 are essential for granule-related genes expression in neutrophil maturation. c-Myb and Runx1 bind to the neutrophil-specific genes promoter and interact to cooperatively regulate their expressions.

From the genetic epistasis and biochemical analysis, it can be concluded that Runx1 functions as a positive regulator for neutrophil maturation in early development. In addition, it interacts and cooperates with c-Myb to transactivate a panel of neutrophil maturation-related genes (Fig. 5).

## Discussion

In this study, by utilizing the zebrafish model, the *in vivo* roles of Runx1 were elucidated in neutrophil maturation. The results demonstrated a cooperative genetic and molecular interaction between Runx1 and c-Myb in regulating neutrophil development. These data give crucial insights into the genetic and molecular orchestration involved in neutrophil maturation.

In hematopoiesis, RUNX1 is known to function in the formation of hematopoietic stem cells (12, 31), the fate of macrophages/neutrophils (14), and the maturation of megakaryocytes and lymphocytes (19, 32, 33). Moreover, RUNX1 is one of the most frequently mutated genes in a variety of hematological malignancies, such as acute myeloid leukemia and familial platelet disorder (34, 35). The high incidence of RUNX1 mutations in multiple types of hematologic malignancies provides strong evidence for its essential function in blood lineage development. At present, more than 50 chromosome translocations that affect RUNX1 function have been identified, most of which cause maturation arrest of myeloid cells and even leukemia (36). Recent studies have shown that RUNX1 is part of a transcriptional complex that regulates important target genes in myelopoiesis. The key questions that remain to be answered are precisely what mechanisms and target genes underlie myelopoiesis and leukemogenesis and how they can be used for developing potential targeted therapies for these patients. From previous mouse studies, the precise role of RUNX1 in primitive myelopoiesis is still unknown. The present study provides direct evidence of a novel function of Runx1 and its regulatory mechanism in the primitive neutrophil maturation process. The clarification of *in vivo* roles of *Runx1* in early myeloid cell development will shed new light on a better understanding of RUNX1-related hematological diseases.

Neutrophil maturation requires a set of neutrophil granule-related products that are gradually produced while granules are properly assembled. The lack of granule contents will lead to immature or dysfunctional neutrophils and ultimately immunodeficiency. In *c-myb* and *runx1* mutants, neutrophil granule formation was severely blocked. In addition, granzyme genes were inhibited as well as an observed downregulation of the granule sorting and packing-related proteoglycan gene, *srgn*. These results underscored the interplay of c-Myb and Runx1 cooperatively controlling neutrophil development by transactivating a cluster of neutrophil maturation-related genes. It was also reported that the ETS family, C/ebp family, and Runx family of transcription factors all function in forming comprehensive genetic networks in myelopoiesis (37, 38, 39, 40). Thus, how myeloid-lineage-specific genes are regulated by different transcription factors is of great interest for future studies. In addition, precisely why these transcription factor pairs function in neutrophils remains to be determined. It is possible that during neutrophil development, transcription factors might be sequentially expressed in different subpopulations of developing neutrophils and/or they might be activated by the same factor. It will be important to derive a comprehensive target gene list of c-Myb and Runx1. In addition, it is crucial to find upstream factors and interacting proteins to elucidate the comprehensive regulation of the neutrophil maturation process.

Taken together, this study has demonstrated the *in vivo* role of Runx1 in neutrophil maturation during early myelopoiesis. Furthermore, a genetic interaction between the two transcription factors, Runx1 and c-Myb, was shown to regulate neutrophil maturation through a molecular interaction that functions to regulate genes expression in a cooperative manner. This study improved our understanding of the genetic networks that orchestrate primitive myeloid cell development and revealed the molecular basis of neutrophil-related disease pathogenesis.

## Experimental procedures

### Zebrafish (*Danio rerio*) strains

Zebrafish *runx1* (Gene ID: 58126) and *c-myb* (Gene ID: 30519) were used in the study. The following strains were utilized: AB, *Tg(mpx:GFP)*i114 (26), *runx1*^*w84x*^ (22), and *c-myb*^*hkz3*^ (23). *runx1*^*w84x*^ mutant harbors a G to A nucleotide substitution resulting a premature truncation in the Runt domain of the Runx1 protein. This truncation removes most of the residues important in Runx1 activity, such as CBFb and DNA binding, and nuclear localization signal. *c-myb*^*hkz3*^ mutant harbors a splice mutation that results in the synthesis of a truncated c-Myb protein lacking its transactivation domain. Zebrafish were maintained in accordance with the Guidelines from the Animal Care and Use Committee of South China University of Technology. All experimental protocols were approved by the Division of Cell, Developmental and Integrative Biology, School of Medicine, South China University of Technology.

### May–Grünwald–Giemsa staining of embryonic blood cells

Fish embryos were anesthetized in 90% PBS + 10% FBS containing 0.02% tricaine. After tail clipping using syringe needle, blood cells were collected by pipetting and cytospun onto slides by centrifugation at 450 rpm for 3 min using a Cytospin 4 (Thermo Scientific). The slides were then airdried and subjected to May–Grünwald–Giemsa (Merk) staining according to the standard protocol. Each group was collected from ∼150 embryos.

### Histology and heat-shock-inducible experiment

WISH and SB staining were performed at 36 hpf onward according to previous report (24, 41), as primitive neutrophils were evident for detection. Relative ratio of SB signal intensity was calculated by Image J software. Heat-shock-inducible constructs (hsp70:Myc-*c-myb* and hsp70: Myc-*runx1*) were generated by inserting Myc-*c-myb* and Myc-*runx1* into the pTol vectors under the control of the heat shock protein 70 (hsp70) promoter. Embryos injected with plasmids were incubated at 39.5 °C for 1 h heat shock treatment at 24 hpf.

### Cell transfection, immunoprecipitation, and western blot

293T cells were grown in DMEM supplemented with 10% bovine calf serum. Cell transfection, cell extracts preparation, immunoprecipitation, and western Blot have been described (42). Constructs with Myc-tagged zebrafish *c-myb* and Flag-tagged zebrafish *runx1* were transfected into 293T cells. Cell lysates were immunoprecipitated with anti-FLAG antibody, and the immunoprecipitants were examined by western blot using anti-MYC and anti-FLAG antibodies. Input represents 10% of total cell lysates used for immunoprecipitation. Anti-MYC and anti-FLAG antibodies were obtained from Santa Cruz (sc-40) and Sigma (ab6658), respectively.

### Microscopy and imaging

VE DIC microscopy was done with 60×/1.00 NA water-immersion objective mounted on Nikon 90i microscope according to previously described (25). In 2-dpf live embryos, the neutrophil granule status is easy to be detected with DIC assay since the neutrophil granules are abundant and neutrophils are accessible to be observed by the lens.

### ChIP assay

Putative transcriptional binding sites were identified by JASPAR online software. Embryos were injected with *Myc*-*c-myb* or *Myc*-*runx1* plasmids at one-cell stage. Six hundred injected embryos were harvested at 2 dpf for brief fixation. Cross-linked chromatin was immunoprecipitated with anti-Myc antibody or anti-IgG antibody (negative control) according to the procedure described by Hart *et al.* (43). The immunoprecipitants were subjected to semiquantitative PCR. ChIP primers could be found in Table S1.

### Bacterial killing experiment

The Tg(mpx:GFP) background zebrafish were anaesthetized at 2 dpf with tricaine (MS-222; Sigma-Aldrich) and injected in the tail muscle with approximately 200 c. f. u. dsRed-labled *E. coli*. Embryos were anaesthetized with tricaine and mounted in 1% (w/v) low-melting-point agarose for time-lapse microscopy. Bacterial clearance time in mpx:GFP^+^ neutrophils was counted.

### Statistical analysis

Data were analyzed by GraphPad Prism6 software using the Student *t*-test for comparisons between two groups and one-way analysis of variance (ANOVA; with Tukey posttest adjustment) among multiple groups. Significance was accepted when *p* < 0.05. Data are expressed as mean ±standard error of the mean (SEM). An asterisk indicates a statistical difference (∗*p* ≤ 0.05, ∗∗*p* ≤ 0.01, ∗∗∗*p* ≤ 0.001, ∗∗∗∗*p* ≤ 0.0001).

## Availability of data and materials

All data generated or analyzed during this study are included in this published article [and its supplementary information files].

## Conflict of interest

The authors declare no competing or financial interests.
