# Supplementary material for: Runx1 regulates zebrafish neutrophil maturation via synergistic interaction with c-Myb
Source: J Biol Chem. 2021 Jan 9;296:100272. doi: 10.1016/j.jbc.2021.100272 (PMC7948814; doi:10.1016/j.jbc.2021.100272)

**Runx1 regulates zebrafish neutrophil maturation via synergistic interaction with c-Myb**

Zhibin Huang^1,#^, Kemin Chen^1,#^, Yali Chi^2^, Hao Jin^3^, Li Li^4^, Wenqing Zhang^1^, Jin Xu^1^, Yiyue Zhang^1,*^

**List of the material**

Figure S1

Figure S2

Figure S3

Table S1

**
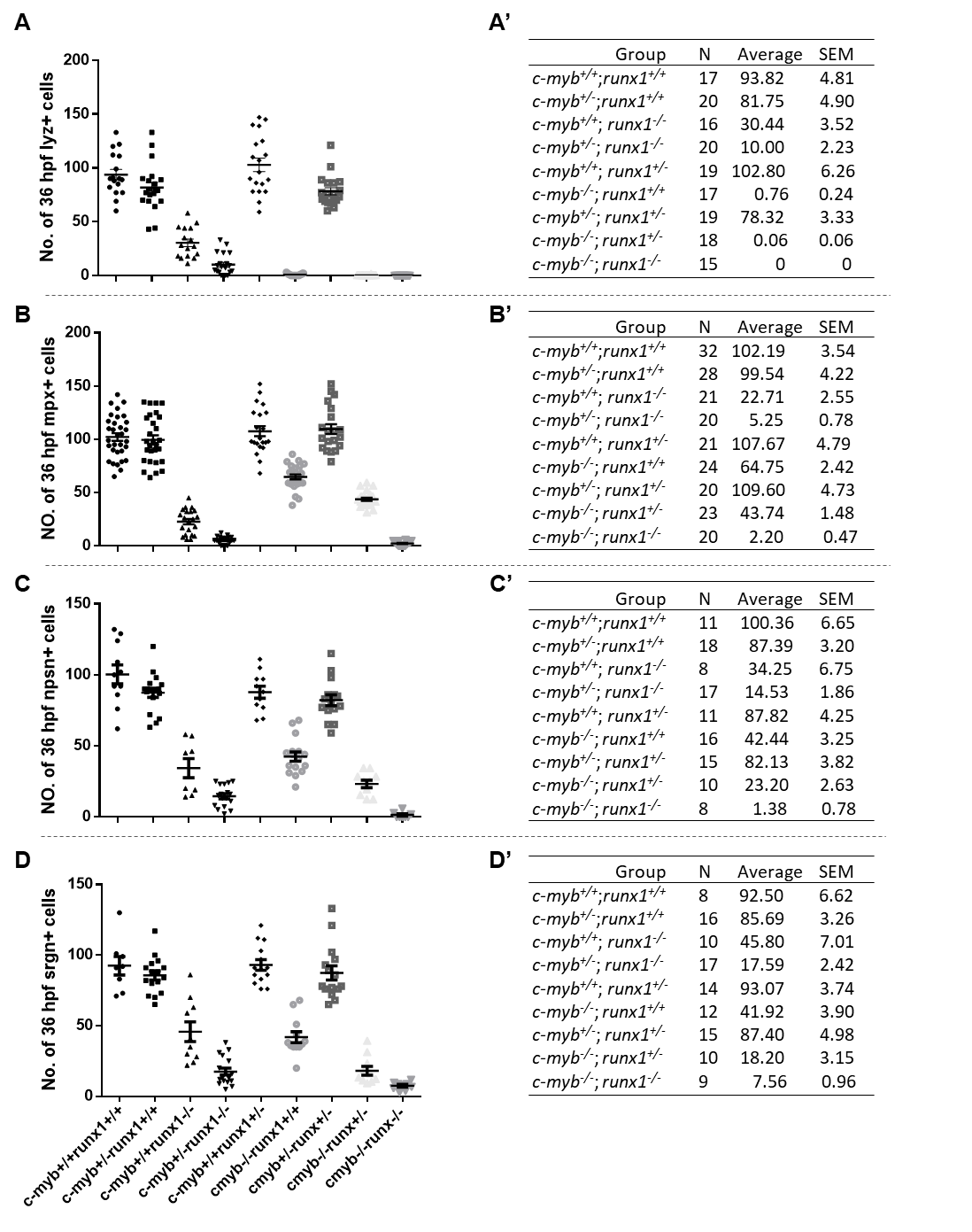
Figure S1 Neutrophil maturation-related genes expression in c-Myb and Runx1 double mutants.**

Quantifications of *lyz*^+^ (A and A’), *mpx*^+^ (B and B’), *npsn*^+^ (C and C’) and *srgn*^+^ (D and D’) cells between 36-hpf *c-myb*^+/-^;*runx1*^+/-^ intercrossed embryos.


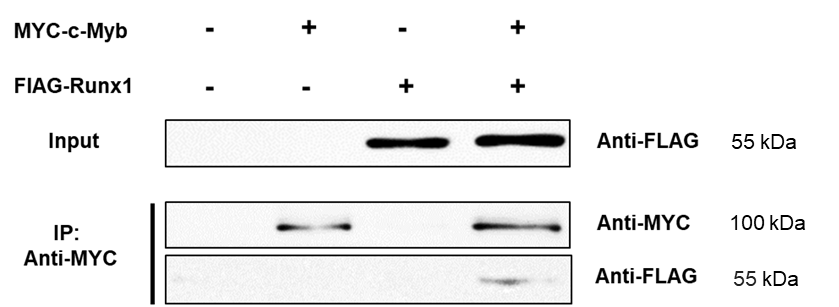


**Figure S2 *In vivo* co-immunoprecipitation experiment in zebrafish embryos.**

Embryos overexpressed with *Myc*-tagged *c-myb*, and *Flag*-tagged *runx1* were immunoprecipitated with anti-MYC antibody. The immunoprecipitates were subjected to western blotting with anti-MYC and anti-FLAG antibody.

**
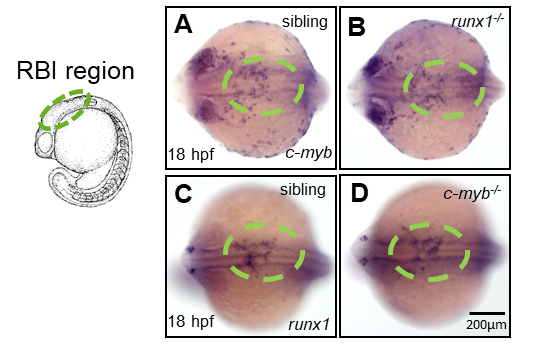
**

**Figure S3** **The independence of c-myb and runx1 transcriptional regulation.**

At the onset of the neutrophil program at the RBI region, WISH showed comparable *c-myb* expression in 18-hpf siblings (E) and *runx1^-/-^* mutants (F). (G-H) Unaltered *runx1* expression in 18-hpf siblings (G) and *c-myb^-/-^* mutants (H). Green circles indicate *c-myb*^+^ and *runx1*^+^ neutrophils progenitors in each panel. Embryos are viewed dorsally with the anterior to the left. (each n≥20)


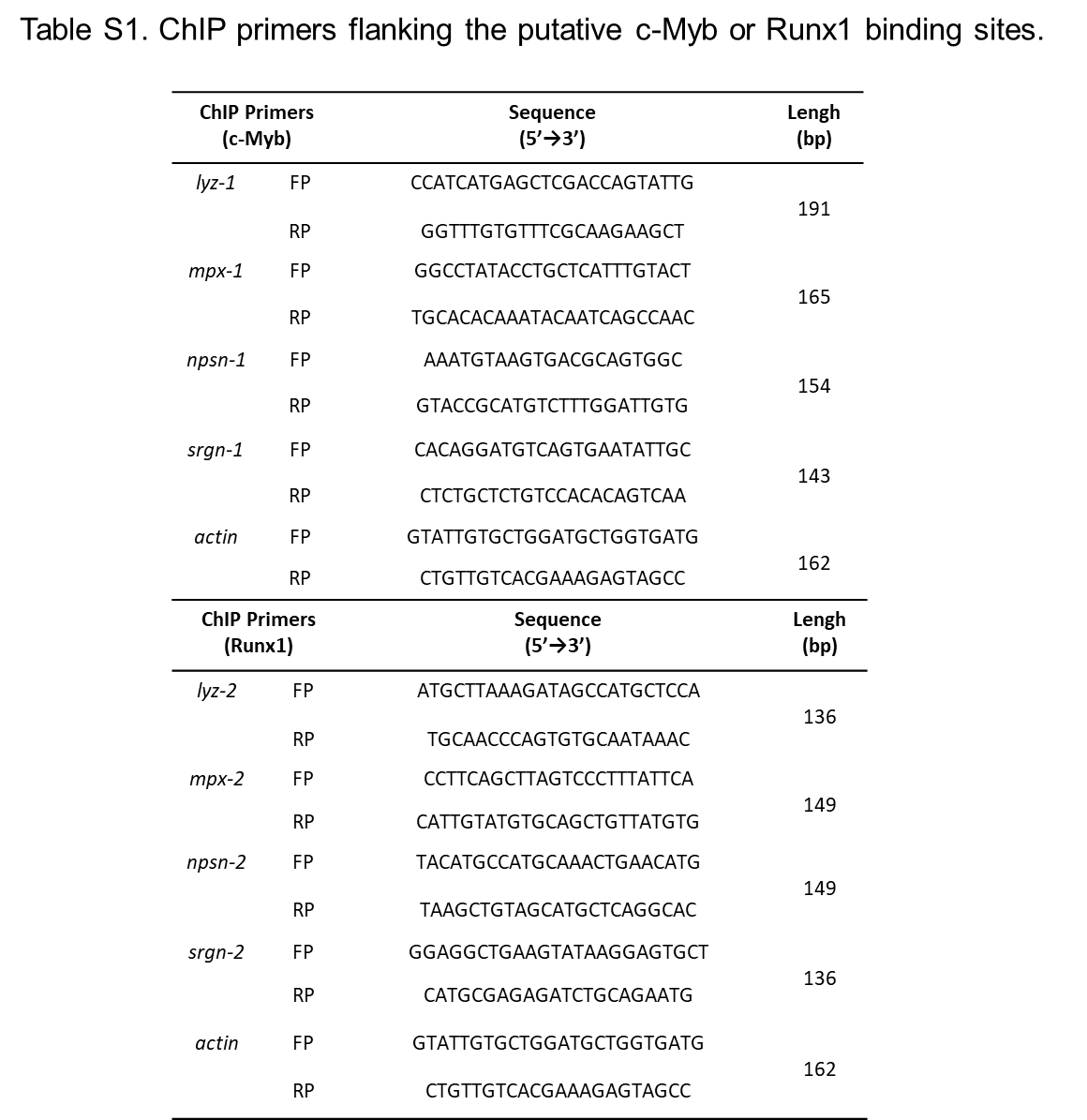

Supplement: Supplemental Figures and Table [file mmc1.docx]
